# Protein Profile of Multiple Myeloma‐Derived Extracellular Vesicles for the Discovery of Novel Myeloma‐Related Biomarkers

**DOI:** 10.1111/cas.70473

**Published:** 2026-07-16

**Authors:** Natalia Platonova, Gilda Aiello, Domenica Ronchetti, Valentina Citro, Rosanna Asselta, Lavinia Casati, Domenica Giannandrea, Elisa Taiana, Antonino Neri, Gloria Sini, Elisabetta Calzavara, Mauro Turrini, Alfonsina D'Amato, Raffaella Chiaramonte

**Affiliations:** ^1^ Department of Health Sciences University of Milan Milan Italy; ^2^ Department of Human Science and Quality of Life Promotion Telematic University San Raffaele Rome Italy; ^3^ Department of Oncology and Hemato‐Oncology University of Milan Milan Italy; ^4^ Department of Biomedical Sciences Humanitas University Pieve Emanuele Milan Italy; ^5^ IRCCS Humanitas Research Hospital Rozzano Milan Italy; ^6^ Hematology Unit Fondazione IRCCS Ca' Granda Ospedale Maggiore Policlinico Milan Italy; ^7^ Scientific Directorate Azienda USL‐IRCCS di Reggio Emilia Reggio Emilia Italy; ^8^ Hematology Unit Valduce Hospital Como Italy; ^9^ Department of Pharmaceutical Sciences University of Milan Milan Italy

**Keywords:** biomarkers, extracellular vesicles, migration inhibitory factor, multiple myeloma, proteomics

## Abstract

Despite therapeutic advances, multiple myeloma (MM) remains characterized by progression and relapse, underscoring the need for reliable biomarkers for early detection and monitoring. Extracellular vesicles (EV) are cell‐derived, lipid bilayer–enclosed particles that mediate intercellular communication and circulate in body fluids, making them promising biomarkers. This study compares the proteome of MM‐derived EV with parental cells and evaluates whether EV represent a superior source of MM‐specific biomarkers. We used high‐resolution mass spectrometry to analyze MM cell lines and their EV. Cell line–derived EV offer a cleaner, tumor‐specific protein profile, minimizing background from nonmalignant cells and improving biomarker accuracy. MS datasets were subjected to bioinformatic analysis, followed by functional enrichment using gene set libraries such as Gene Ontology, Jensen Compartments, TRRUST and DisGeNET, to extend interpretation beyond limited protein databases. Compared to cellular proteins, the EV proteome was enriched in functions related to oncogenic signaling, transcriptional regulation and MM pathophysiology, strengthening its potential as a cancer‐related biomarker source. DisGeNET identified cancer‐related proteins that were further evaluated to identify MM‐specific candidates analyzing MM patients' gene expression databases containing clinical data. This led to the identification of macrophage migration inhibitory factor (MIF) and prohibitin. ELISA on plasma from MM patients detected significantly increased MIF levels in smoldering and active MM compared with healthy donors. Approximately half of circulating MIF was EV‐associated in MM patients, indicating that MIF is distributed between vesicular and soluble plasma fractions and underscoring its potential as a noninvasive biomarker for MM early detection and patient monitoring.

## Graphical abstract

Comparison of extracellular vesicles with parental cells through proteomic profiling, followed by an integrated bioinformatic pipeline including disease association and transcriptomic analyses, identifies candidate biomarkers for multiple myeloma. Among these, MIF emerges as a clinically relevant marker, supported by validation in patient samples and enabling minimally invasive disease monitoring.

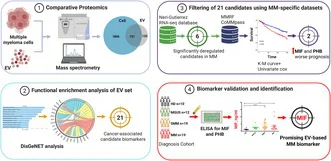

**Abbreviations**
BM: bone marrow
Cryo‐EM: cryo‐electron microscopy
DisGeNET: gene‐disease associations dataset
EV: extracellular vesicles
HD: healthy donors
HMCL: human MM cell lines
MGUS: monoclonal gammopathy of undetermined significance
MIF: macrophage migration inhibitory factor
MM: multiple myeloma
MM‐EV: MM‐derived EV
MMRF: Multiple Myeloma Research Foundation
MS: mass spectrometry
nLC‐HRMS: Nano‐Liquid Chromatography High‐resolution Mass Spectrometry
NTA: Nanoparticle Tracking Analysis
OS: overall survival
PCL: plasma cell leukemia
PFS: progression‐free survival
PHB: prohibitin
SMM: smoldering MM
TRRUST: transcriptional regulatory relationships unraveled by sentence‐based text mining

## 1 Introduction

Multiple myeloma (MM) remains an incurable plasma cell malignancy characterized by the localization of clonal plasma cells in the bone marrow (BM). According to the World Health Organization, MM represents 13% of all cases of hematologic malignancies and 1% of all cancers [1]. MM is characterized by a wide clinical spectrum tumor, ranging from the premalignant condition of monoclonal gammopathy of undetermined significance (MGUS) to asymptomatic smoldering MM (SMM) up to truly overt and symptomatic MM, which finally may progress to the most aggressive form of secondary extra‐medullary plasma cell leukemia (PCL) [1]. Despite therapeutic advances, MM is still incurable due to intrinsic or acquired drug resistance resulting from interaction of MM cells with the BM niche [1]. BM microenvironment comprises various cell types, including stromal, bone, immune, endothelial and stem cells, which interact with tumor cells and promote their growth, survival and chemoresistance [2, 3]. Increasing evidence shows that the crosstalk of MM cells and the BM niche can be mediated by extracellular vesicles (EV) [4].

EV are a heterogeneous population of vesicles classified into two main groups based on their size and biogenesis: small EV (~30–200 nm) or medium/large EV (> 200 nm) [5]. EV influence cell‐to‐cell communication by carrying various biomolecules, such as mRNAs, microRNAs, proteins and lipids. They also represent an optimal source of biomarkers that can be easily collected from blood, a feature on which this group has focused its research efforts in recent years across several diseases. MM‐derived EV (MM‐EV) shape the BM niche, promoting angiogenesis, immune suppression and osteoclastogenesis [6, 7]; in turn, EV derived from BM stromal cells favor MM cell proliferation and drug resistance [8]. Furthermore, EV from MM may play a role in the spread of MM to distant sites, consequently promoting skeletal metastasis formation and bone‐related disorders [9, 10].

Considering their involvement in MM progression, EV and their cargo have been explored as biomarkers for early detection, monitoring of MM disease progression, and clinical features such as drug resistance and treatment monitoring. Recent evidence indicates that miRNA cargo in circulating EV of MM patients could have a prognostic role in MM [11, 12, 13]. Circulating EV from MM patients express tumor‐related antigens and display characteristic size distribution and concentration [14, 15]. High EV protein/particle ratio, or EV cargo > 0.6 μg/10^8^ particles, is related to poorer survival and immune dysfunction in MM patients [16].

Recently, high‐throughput proteomic studies by mass‐spectrometry technique have emerged as a powerful tool in discovering new EV‐related biomarkers in various diseases, including MM. Proteomics studies have shown differential protein content of EV isolated from MM cells, peripheral plasma as well as BM of MM patients [17, 18]. EV‐derived CD44 molecule has been identified as the key molecule with a role as a prognostic marker for MM disease [18].

In this study, we employed a comprehensive proteomic workflow to characterize the protein cargo of EV derived from a multiple myeloma cell line and to compare their composition with that of parental cells. We then integrated these proteomic data with multi‐omics clinical datasets from large MM patient cohorts to identify MM‐EV associated markers with potential diagnostic or prognostic value. Finally, we validated selected candidates in plasma samples from MM patients across disease stages. ELISA validation on MM patients' plasma confirmed the macrophage migration inhibitory factor (MIF) clinical relevance. Half of circulating MIF was EV‐associated and correlated with disease tumor progression toward smoldering and active MM.

## 2 Materials and Methods

### 2.1 Cell Line Culture

Human MM cell lines (HMCL), OPM2 (ACC‐50), JJN3 (ACC‐541) and AMO1 (ACC‐538) were purchased from the German Collection of Microorganisms and Cell Cultures, whereas RPMI8226 (ATCC CCL‐155) and U266 (ATCC TIB‐196) were purchased from the American Type Culture Collection. HMCL were cultured in RPMI1640 (Euroclone srl, Italy) supplemented with 10% FBS (Euroclone srl), 100 U/mL penicillin/streptomycin (Microgem, Italy), and 2 mM L‐glutamine (Microgem), following standard culture conditions.

### 2.2 Isolation of Extracellular Vesicles From HMCL

HMCL were cultured for 48 h in RPMI1640 medium depleted of FBS‐derived bovine EV by ultracentrifugation at 110,000 × g 16 h. Cell culture supernatants were centrifuged at 300 × g for 5 min, the resulting cell pellets were collected for cellular lysates (lysed in 8 M urea buffer) and supernatants underwent sequential centrifugations at increasing speeds to remove debris and aggregates [7]. EV were collected by 75 min ultracentrifugation at 110,000 × g at 4°C using a Himac CP100NX ultracentrifuge (Himac, Japan). EV pellets were resuspended in PBS 1X and ultracentrifugation was repeated under the same conditions. The EV pellet was immediately resuspended in PBS 1X for downstream assays or lysed in 8 M urea buffer for proteomics, then stored at −80°C.

### 2.3 Nanoparticle Tracking Analysis (NTA)

EV preparations were quantified by NTA using a NanoSight Pro instrument (Malvern Panalytical Ltd., UK) equipped with a 488 nm laser. EV were diluted in PBS 1X without Ca^++^ and Mg^++^ (Euroclone, Italy) to reach the optimal concentration. Data acquisition and analysis were conducted using NS Xplorer Software v1.1.0.6 (Malvern Panalytical).

### 2.4 Cryo‐Electron Microscopy

EV morphology was examined by cryo‐electron microscopy (Cryo‐EM) at the Unitech NOLIMITS facility (University of Milan). Approximately 1 × 10^10^ EV in PBS 1X were applied directly onto EM grids, vitrified and imaged using a Talos Arctica microscope (200 kV) equipped with a Ceta 16 M detector. Images were acquired at 28,000× magnification.

### 2.5 Multiplex Flow Cytometry Characterization of EV Tetraspanins

EV surface tetraspanins were detected in EV preparation using the bead‐based multiplex assay MacsPlexEV Kit IO human (Miltenyi Biotec, Italy). Briefly, 2 × 10^9^ EV resuspended in PBS 1X were incubated overnight with fluorescent‐labeled MACSPlex capture beads followed by staining with MACSPlex APC‐conjugated detection antibodies for CD9, CD63, and CD81 according to the manufacturer's instructions. Samples were acquired on a Cytek Aurora Spectral Flow cytometer (Cytek, Italy).

### 2.6 Nano‐Liquid Chromatography High‐Resolution Mass Spectrometry (nLC‐HRMS)

OPM2 cells and matched EV (biological triplicates) were lysed in 8 M urea (50 mM Tris HCl, 30 mM NaCl, pH 8.5) with protease inhibitors. Protein concentration was measured by BCA assay (Pierce BCA, Thermo Fisher Scientific, Italy). For each sample, 10 μg protein resuspended in 50 mM NH_4_2084HCO_3_ were reduced with 5 mM DTT (Sigma‐Aldrich, Italy; 30 min, 52°C, shaking) and alkylated with 15 mM iodoacetamide (Sigma‐Aldrich; 20 min, RT, dark). Proteins were digested with trypsin (Trypsin Sequencing Grade; Roche, Monza, Italy; 1:20, w/w; overnight, 37°C, 650 rpm) [19].

To increase the quality of instrumental analysis, the digested samples were further purified and concentrated by 0.6 μL C‐18 resin ZipTip (Millipore, Italy). Tryptic peptides were analyzed on a Dionex Ultimate 3000 nano‐LC system (USA) connected to an Orbitrap Fusion Tribrid Mass Spectrometer (Thermo Scientific, Germany) equipped with a nano‐electrospray ion source. Peptide mixtures were pre‐concentrated on an Acclaim PepMap 100–100 μm × 2 cm C25 and separated on an EASY‐Spray column, 25 cm × 75 μm ID packed with Thermo Scientific Acclaim PepMap RSLC C18, 3 μm, 100 Å. The temperature was set to 35°C and the flow rate was 300 nL/min. Mobile phases were 0.1% formic acid in water (solvent A), 0.1% formic acid in acetonitrile (solvent B). The elution gradient consisted of 4%–28% B for 90 min and then 28%–40% for 10 min, followed by 95% for the next 6 min to rinse the column [19].

### 2.7 Data Analysis

Raw MS files were processed by the MaxQuant software v1.6.6.0 [20] set on the Uniprot_*Homo sapiens* database against the Andromeda search engine. The Andromeda search engine used to identify MS/MS based peptide and proteins in MaxQuant comprises a target‐decoy approach with less than 1% of False Discovery Rate. Ly‐sC and trypsin as the digestive enzymes, variable modification of carbamidomethylation of cysteine (+57.021 Da), fixed modification of methionine oxidation (+15.995 Da), N‐terminal acetylation (+42.011 Da) were set as further parameters.

Raw data are available in ProteomeXchange (PXD063979) [21]. Venn diagrams of protein identifications were generated with the FunRich software (version 3.1.3). Functional enrichment analyses were performed with Enrichr using selected gene set libraries (Gene Ontology, Jensen Compartments, TRRUST, DisGeNET) as follows: https://maayanlab.cloud/Enrichr [22].

### 2.8 Multi‐Omics Data in CoMMpass Study

Multi‐omics data about BM MM samples at baseline (BM_1) were publicly accessible from MMRF CoMMpass Study (https://research.themmrf.org/) including more than 1000 MM patients from several worldwide sites and retrieved from the Interim Analysis 20 (MMRF_CoMMpass_IA20, accessed on 19 January 2023). Transcript per Million reads values of the investigated transcripts were retrieved using Salmon gene expression quantification data (MMRF_CoMMpass_IA20_salmon_geneUnstranded_TPM) in 767 BM_1 MM patients.

Overall survival (OS) and progression‐free survival (PFS) clinical data were analyzed for 753 MM patients for which both RNA‐seq expression and survival information were available. Non‐synonymous somatic mutation variants and counts data were obtained from whole‐exome sequencing analyses, main IgH translocations were inferred from RNA‐seq spike expression estimates of known target genes and Copy Number Alteration data were retrieved by means of Next‐generation Sequencing‐based FISH [23] in 489 MM cases for which all data were available [24]. The presence of a specific copy number alteration was considered when it occurred in at least one of the investigated cytoband at a 20% cut‐off for each considered chromosomal aberration, as previously reported [24]. Survival analyses were performed as previously described [25].

### 2.9 MM Patients

Peripheral blood was collected at diagnosis from newly diagnosed MM patients and age−/gender‐matched healthy donors (HD). The study was approved by the Institutional Review Board of Insubria (Italy) (protocol 285/2023, 14/06/2023). Written informed consent was obtained in accordance with the Helsinki Declaration. Disease status was defined as MGUS, SMM, or active multiple myeloma (MM) according to International Myeloma Working Group guidelines [26]. Main clinical information of patients is reported in Table S1. Samples were collected in EDTA tubes, centrifuged at 250 × *g* for 20 min at room temperature, and plasma aliquots were stored at −80°C.

### 2.10 Western Blot Analysis

Western blots were performed on cellular and EV lysates as described previously [27]. In brief, 20 μg of protein per sample were resolved on 15% SDS‐PAGE and transferred to nitrocellulose (Hybond‐ECL; Amersham Bioscience, Italy). Membranes were blocked in 5% nonfat milk in TBS‐T and probed overnight (4°C) with primary antibodies against ITGβ1 (1:4000, GTX128839; GenTex, USA), Flotillin2 (1:500, GTX114411; GenTex), DNMT1 (1:1000, sc‐20701; Santa‐Cruz Biotechnology, USA), HDAC2 (1:1000, sc‐7899; Santa‐Cruz Biotechnology), CDK6 (1:1000, D4S8S; Cell Signaling Technology, USA), Annexin11 (1:3000, ab300578; Abcam, USA), Histone 3 (1:5000, ab1791; Abcam), HSP90α (1:10000, GenTex), PHB (1:1000, A19530, Abclonal, Germany) and MIF (1:500, A22623; Abclonal). HRP‐conjugated secondary antibodies (goat anti‐rabbit; Millipore) and Western Bright ECL (Advansta, USA) were used for detection on a ChemiDoc Imaging System (Bio‐Rad, USA). Western blot analyses were repeated at least two times.

### 2.11 ELISA

ELISA for MIF (Abclonal, Human MIF ELISA kit, n. RK00125, Germany) and PHB (FineTest, Human PHB ELISA kit, n. EH3566, China) content in plasma was performed according to the manufacturers' instructions. Plasma was diluted 1:10 for MIF and 1:5 for PHB; all samples were assayed in triplicate. Absorbance at 450 nm was recorded on an Ensight plate reader (PerkinElmer, Italy) and concentrations were derived using CurveExpert 1.4.

### 2.12 Statistical Analysis

Data were analyzed using ANOVA, with Tukey's post hoc test applied for pairwise multiple comparisons.

## 3 Results

Emerging evidence indicates that EV are key players in cancer cell communication and tumor progression and represent a potential source of diagnostic and prognostic biomarkers. This work is based on evidence that EV selectively package their cargo, often leading to the enrichment of specific molecules—including proteins—that reflect the physiological state of the originating cell.

Starting from the hypothesis that selectively enriched components may have a significant potential as biomarkers for early disease detection, prognosis and monitoring treatment responses, this study: (i) investigates if the proteomic cargo of MM‐EV differs from the overall protein content of the parental cells; (ii) examines whether the selectively enriched protein cargo of MM‐EV could serve as a potential source of predictive biomarkers; (iii) validates the candidate biomarkers on MM patients' plasma. To do this, we conducted an in‐depth proteomic analysis of the OPM2 cell line and their shed EV.

### 3.1 Proteomic Profiling Reveals Shared and Distinct Protein Signatures in OPM2 Cells and Derived EV

We first verified the integrity and identity of EV shed by OPM2 cells. Cryo EM confirmed the expected spherical morphology with a clearly delineated lipid bilayer (Figure 1A), while NTA indicated a particle size distribution consistent with small/medium EV (Figure 1B). Multiplex bead–based flow cytometry showed high expression of CD81 and CD63 on OPM2 EV, with lower levels of CD9 (Figure S1).

**FIGURE 1:**
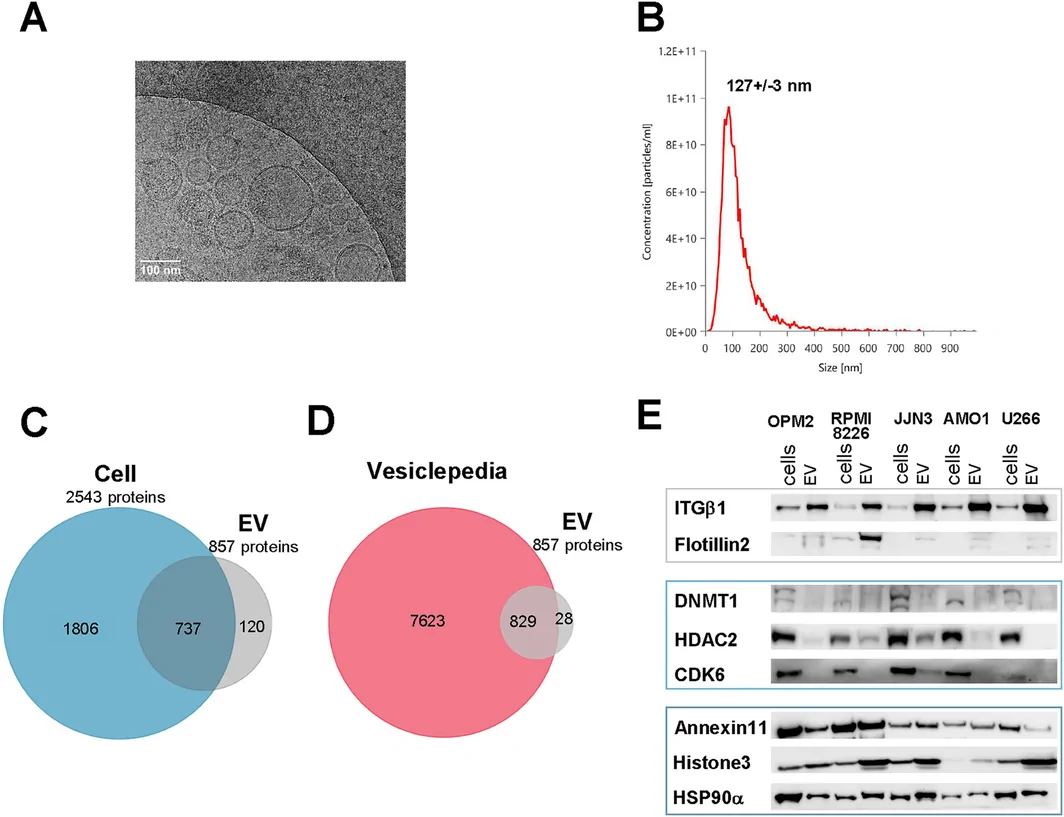
Validation of protein identifications from the proteomic analysis of OPM2 derived EV and cell lysates. (A) Representative cryo‐EM image shows EV in their native state. Scale bar is indicated. (B) Representative result of NTA conducted on EV isolates. The mean particle size (nm) is indicated in the graph. (C) Venn diagram of mass spectrometry data of proteins shared by OPM2 cells and OPM2‐EV. (D) Venn diagram of enrichment of OPM2‐EV proteins compared to Vesiclepedia database using FunRich Software (V3.1.3). (E) Western blot analysis of cell and vesicular lysates from a panel of 5 MM cell lines confirms the enrichment of some representative proteins in EV (gray square), in OPM2 cells (blue square) or shared by both (dark blue square). 20 μg of protein lysates were loaded on lane.

OPM2 cell lysates and the corresponding EV lysates were analyzed by nLC‐HRMS. The proteomic analysis identified 2543 proteins in OPM2 cell extracts (total cellular proteins) and 857 proteins in OPM2‐EV extracts (total EV proteins) (Table S2). Of these, 737 proteins were shared by cells and EV (29.0% of total cellular proteins; 86.0% of EV proteins), 120 proteins were uniquely detected in EV (EV specific, 14.0% of total EV proteins) and 1806 were cell specific (71.0% of cellular proteins) (Figure 1C). Notably, the percentage of EV‐specific proteins and cell‐specific proteins is consistent with previous findings [18]. In addition, 96.7% of the EV protein set (829/857) overlapped with entries deposited in Vesiclepedia, underscoring the robustness of our isolation and proteomic workflow (Figure 1D).

The enrichment of EV‐ or cell‐specific proteins was validated by Western blot for selected proteins on a panel of five HMCL (OPM2, RPMI8226, JJN3, AMO1 and U266). ITGβ1 and Flotillin 2 were enriched in EV lysates; DNMT1, HDAC2 and CD6 resulted abundant in cellular lysates (Figure 1E). Conversely, Annexin 11, Histone3 and HSP90 were similarly expressed in both EV and cellular extracts, confirming MS analysis. Together, these results indicate that MM EV package a selective and biologically meaningful subset of proteins rather than passively mirroring the cellular proteome.

### 3.2 Functional Divergence Between Proteins From OPM2 Cells and Shed EV

To assess whether the selective enrichment of specific proteins in EV and cell extracts correlates with distinct functions, we compared proteins and pathways expressed in OPM2 cells and the shed EV using Enrichr (https://maayanlab.cloud/Enrichr/) on gene set libraries. Our rationale for including gene set databases, despite the primary focus on proteins, was to expand the functional interpretation beyond the scope of limited protein‐centric resources.

Gene Ontology analysis revealed that EV‐specific molecules were strongly associated with extracellular communication and vesicle‐mediated interactions, including cadherin/kinase/GTP binding and localization to focal adhesions and cell–substrate junctions, underscoring their role in cell adhesion and signaling (Figure 2A). In contrast, cell‐specific proteins were enriched in components linked to intracellular regulation, notably RNA metabolism and processing, with functions such as RNA binding and localization within the nucleus and mitochondria, supporting transcription, translation, and energy production (Figure 2B). Subcellular mapping using the Jensen Compartments library (https://compartments.jensenlab.org/) further supported this dichotomy with EV‐specific lists enriched for extracellular exosome/vesicle terms and cell‐specific lists mapping to intracellular compartments (Figure S2).

**FIGURE 2:**
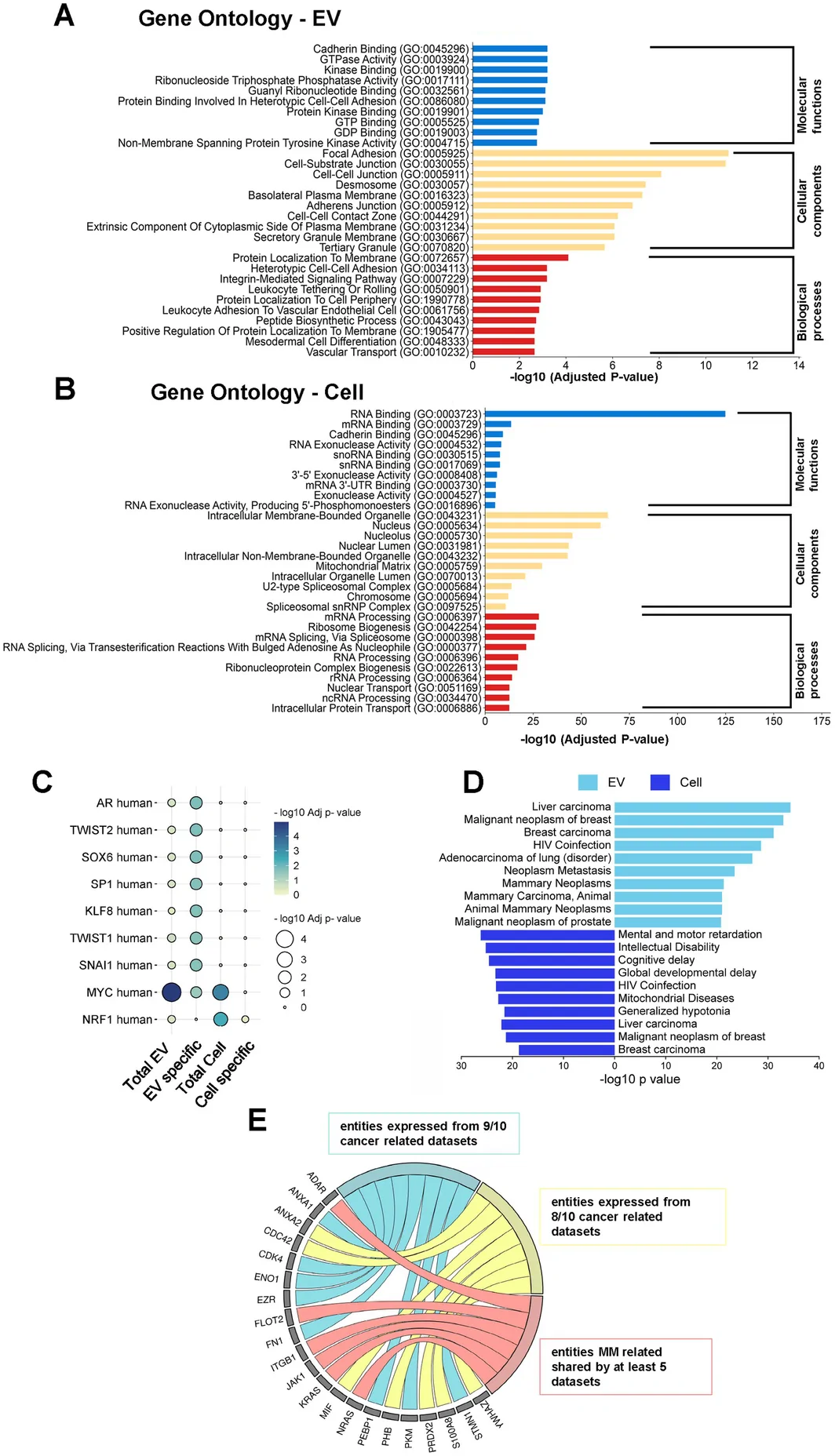
Bioinformatic analysis of proteomic data from OPM2 cells and OPM2‐EV (A, B) Gene Ontology (GO) enrichment analysis detected the top 10 list of statistically significantly enriched terms in biological processes, cellular components and molecular functions for the EV‐specific set (A) and the Cell‐specific set (B). Bar charts display enriched terms ranked by −log_10_(adjusted *p*‐value); the top‐ranked term indicates the strongest enrichment within the input query gene set. (C) Transcriptional regulatory network enrichment analysis using the TRRUST database. Dot plots compare total EV (857 molecules), total Cell (2543 molecules), EV‐specific (120 molecules) and Cell‐specific (1806 molecules) datasets. The color gradient represents the adjusted *p*‐value (−log_10_), with most transcription factors showing significant enrichment in EV specific set proteins. (D) DisGeNET disease enrichment analysis of total EV (light blue) and total Cell (blue) datasets. Bar plots show the top 10 significantly enriched disease‐associated terms ranked by −log_10_(*p*‐value). (E) Chord diagram illustrating 21 molecular entities identified in OPM2‐derived extracellular vesicles through DisGeNET analysis. These entities are present in 5–9 out of 10 tumor‐associated gene sets or show a strong association with multiple myeloma pathology (complete list in Table S3).

Given our prior evidence that MM‐EV transfer NOTCH2 to the tumor microenvironment cell populations [7]—potentially mediating transcriptional regulatory networks—we interrogated transcriptional network representation using TRRUST (Transcriptional regulatory relationships unraveled by sentence‐based text mining) database. The enrichment analysis among the cell and EV proteins detected that EV cargo contains mediators involved in regulation of gene expression, reflecting the aberrant transcriptional activity of the parental MM cells. Strikingly, the MYC oncogenic circuit emerged as the top‐scoring network within EV cargo—even more represented than in parental cells—together with additional networks (AR, TWIST1/2, SOX6, SP1, KLF8 and SNAI1) over represented in the EV specific set (Figure 2C) [28]. These findings suggest that MM EV may transport transcriptional regulators from the tumor cells to modulate the gene expression in nearby and distant target cells.

To explore potential disease associations in EV and cell molecular profiles, we performed DisGeNET enrichment [29]. Among EV molecules, 9 of the top 10 enriched terms were cancer‐related, with prominent associations to liver carcinoma, malignant neoplasms and breast carcinoma; conversely, the cellular set showed a broader representation of non‐oncological disease categories, with only two malignant neoplasm terms within the top results (Figure 2D). Overall, these analyses reinforce the view that MM EV act as a selective reservoir of tumor‐related molecules and transcription factors linked to oncogenic pathways and intercellular signaling and may serve as a significant source of specific tumor biomarkers.

### 3.3 Selection of Potential Predictive Biomarkers: High *MIF* and *PHB* Expression in MM Correlates With Reduced Survival

To identify potential MM EV biomarkers, a set of promising cancer‐related biomarker proteins was identified as recurrently involved across multiple cancer gene datasets within DisGeNET database, based on the premise that commonly shared proteins may possess greater predictive potential. This included: (i) EV molecules shared by all 9 top‐scoring cancer‐related gene sets—i.e. ANXA1, CDK4, ENO1, EZR, FN1, PEBP1, PKM and STMN1; (ii) EV molecules shared by 8 out of 9 cancer gene sets (ANXA2, CDC42, MIF, PHB, PRDX2, S100A8 and YWHAZ); and (iii) EV molecules shared by at least 5 out of 9 cancer gene datasets with established roles in MM‐ JAK1 [30], KRAS [31], ITGB1 [32], NRAS [33], ADAR [34] and FLOT2 [35] (Figure 2E; Table S3).

To explore clinical relevance of the selected 21 genes in MM, we applied a stepwise analytical workflow to identify potential EV‐related biomarkers (Figure 3). 21 proteins selected on their recurrence across disease‐associated datasets using the DisGeNET database were further evaluated through integration with MM‐specific transcriptomic datasets, including those curated by Neri and Gutierrez as well as the MMRF CoMMpass cohort.

**FIGURE 3:**
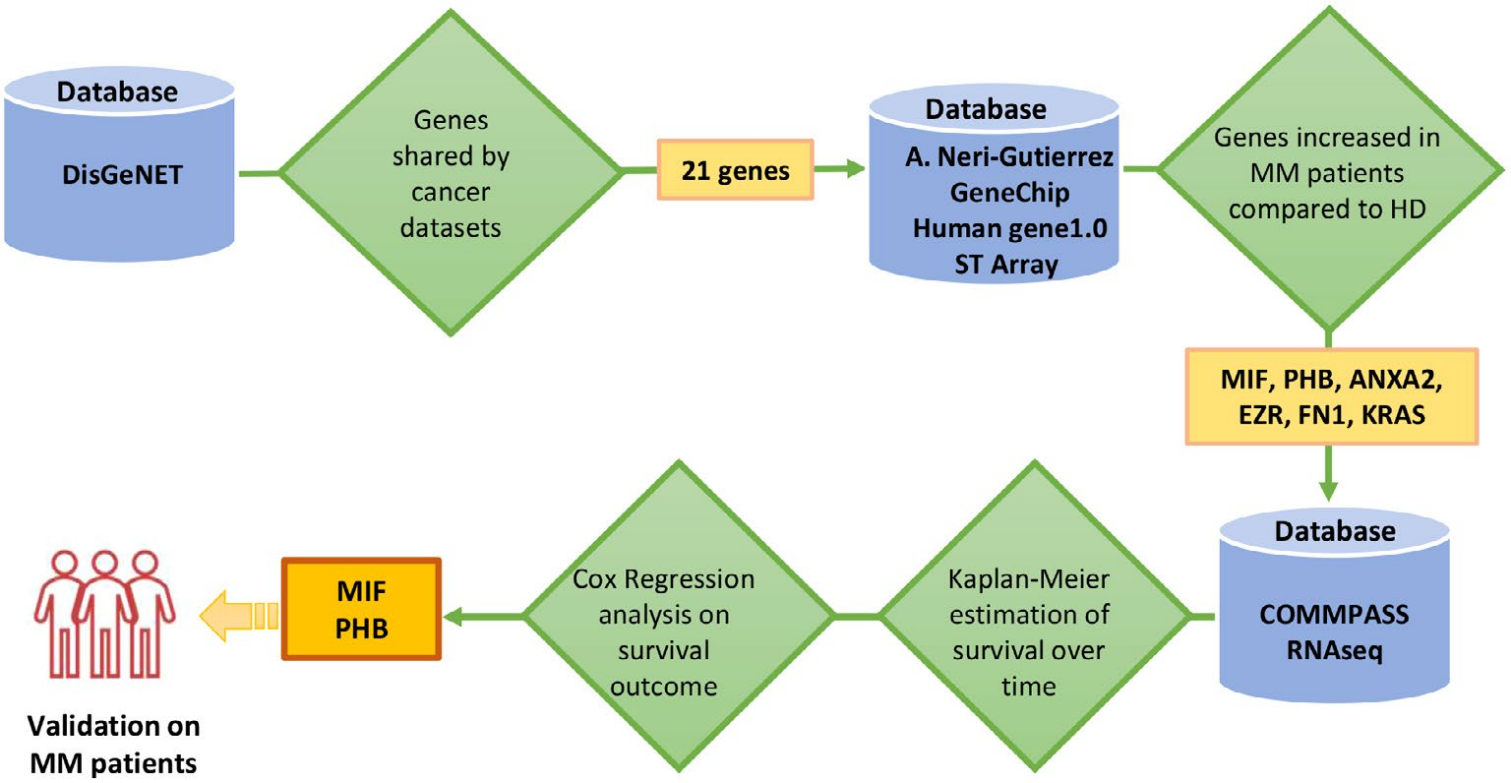
Bioinformatic pipeline for EV‐related biomarker identification. To identify candidate MM EV–associated biomarkers, we applied a stepwise analytical workflow starting from functional enrichment and disease association analysis using the DisGeNET database to prioritize candidates with potential pathological relevance. Selected molecules were subsequently filtered based on their recurrence across disease‐associated datasets, leading to the identification of 21 candidate proteins. MM‐specific validation of these candidates was performed by evaluating them through MM‐specific transcriptomic datasets, including those curated by Neri and Gutierrez, followed by analysis of the MMRF CoMMpass cohort. Analysis through the database curated by Neri and Gutierrez (*n* = 268 MM patients, 9 healthy donors and 18 HMCL) identified six molecules significantly deregulated in newly diagnosed MM compared with healthy donors, namely MIF, PHB, ANXA2, EZR, FN1 and KRAS. To assess their clinical relevance and prognostic impact, we analyzed the MMRF CoMMpass dataset (*n* = 489 MM patients). Kaplan–Meier and Cox regression analyses revealed that high expression levels of MIF and PHB were significantly associated with shorter overall survival (OS) and progression‐free survival (PFS). Finally, selected candidate biomarkers were validated ex vivo in patient plasma samples by ELISA.

In particular, variations in MM patients' gene transcription levels were assessed using the database curated by A. Neri and N. Gutierrez (GSE66293 and GSE47552) including 268 MM samples derived from CD138^+^ cells obtained from BM aspirates and 18 samples derived from HMCL (Figure 4). The MM cohort comprised 9 healthy donors (*N*), 20 MGUS, 33 SMM, 170 MM, 24 primary PCL, 12 secondary PCL patients was analyzed using the GeneChip Human Gene 1.0 ST Array. Six genes—macrophage migration inhibitory factor (*MIF*), Prohibitin (*PHB*), Annexin A2 (*ANXA2*), Ezrin (*EZR*), Fibronectin1 (*FN1*) and Kirsten rat sarcoma (*KRAS*)—were significantly deregulated in newly diagnosed MM versus healthy donors (*MIF*: *p* = 0.0046; *PHB*: *p* = 0.0109, *KRAS p* = 0.0051; *ANXA2 p* = 0.011; *FN1*, *p* = 0.0228; *EZR*, *p* = 0.01) (Figure 4).

**FIGURE 4:**
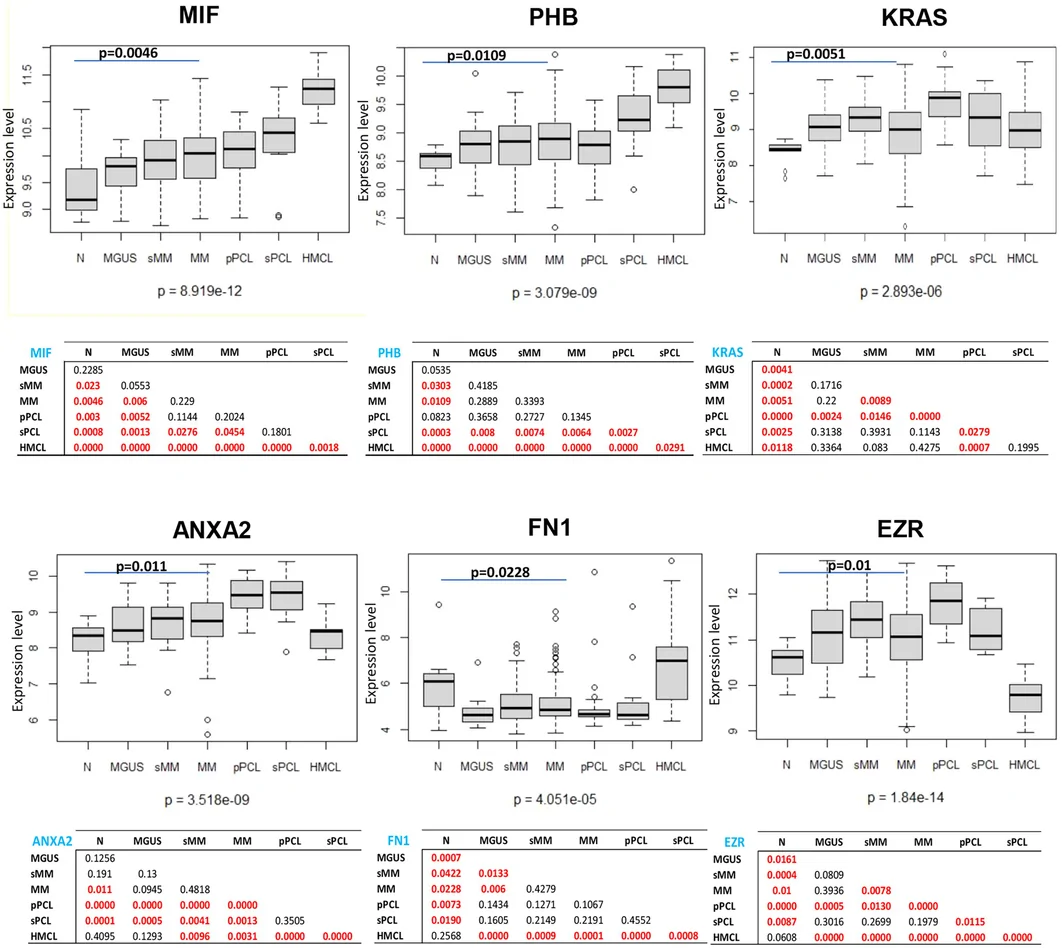
Differential expression of selected genes in MM patients. Box plots illustrate the mRNA expression levels of selected genes in 268 MM samples including 9 normal controls (*N*), 20 monoclonal gammopathy of undetermined significance (MGUS), 33 smoldering multiple myeloma (SMM), 170 multiple myeloma (MM), 24 primary PCL (pPCL), 12 secondary PCL (sPCL), as well as 18 samples from HMCL evaluated by GeneChip Human gene1.0 ST Array. Log values of normalized raw data are reported on the *y*‐axis in the graph. Statistical significance among groups was calculated with Kruskal–Wallis rank sum test (*p*‐values are indicated below each table). Pairwise comparisons (Dunn test) are reported in the accompanying table, with significant *p*‐values highlighted in red.

Further, taking advantage of the MMRF CoMMpass global dataset (*n* = 753 with RNA seq profiles and medical/clinical data) (MMRF_CoMMpass_IA20, accessed on 19 January 2023), we showed that high expression of MIF and PHB was associated with shorter OS and PFS by Kaplan–Meier analysis (Figure 5A). In a subset with complete covariates (*n* = 489), univariate Cox confirmed prognostic associations for MIF and PHB, while multivariate models—adjusted for clinical and genomic predictors—did not retain them as independent variables (Figure 5B; Figure S3). These results position MIF and PHB as biologically compelling, EV‐linked candidates with measurable clinical relevance, warranting orthogonal validation at the protein level.

**FIGURE 5:**
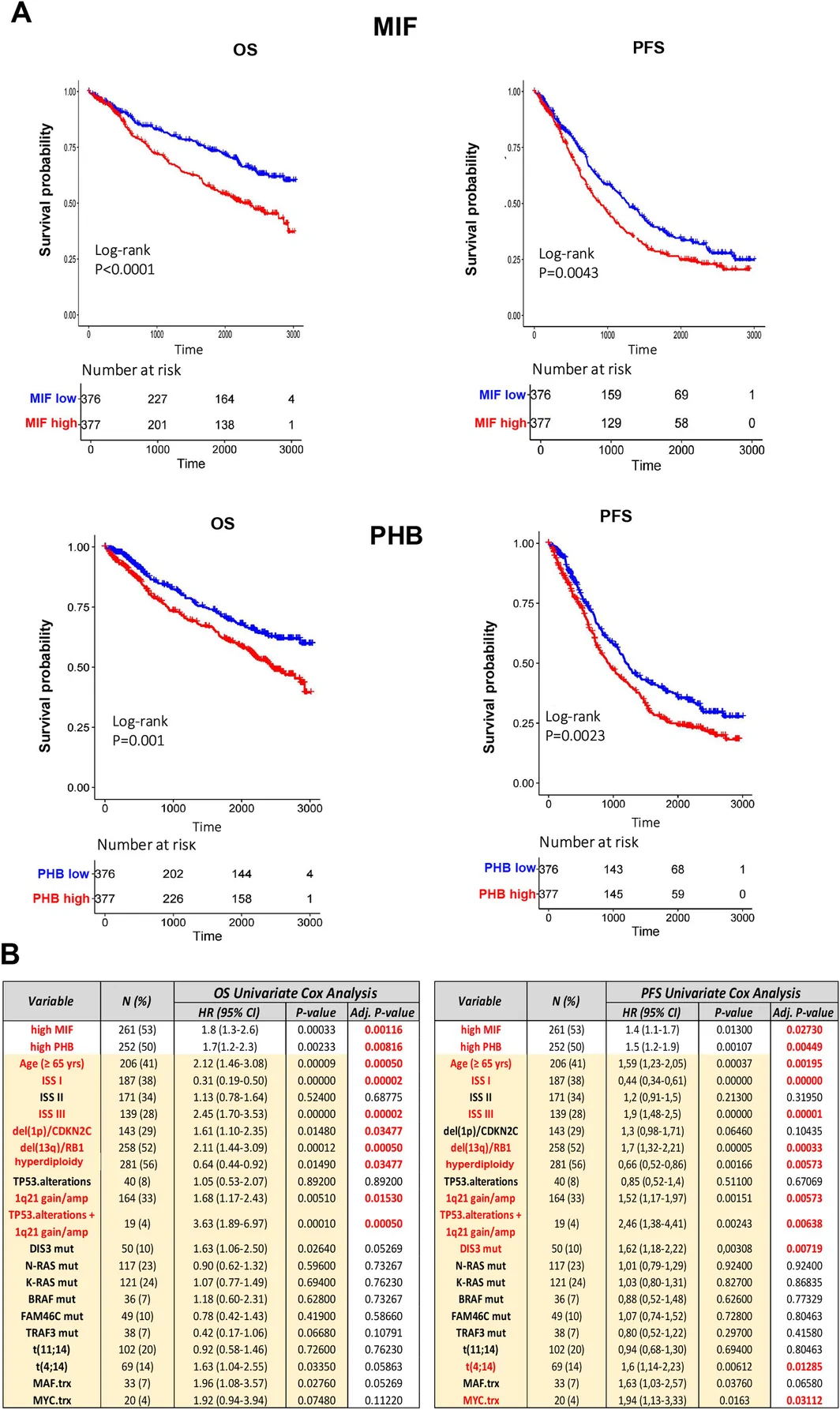
Evaluation of clinical significance of the gene's variability using MMRF CoMMpass Study. (A) Kaplan–Meier curves demonstrate the difference in overall survival (OS) and progress free survival (PFS) among 753 patients with MM stratified in high‐ and low‐expression groups based on the median expression level across the entire dataset for MIF (left) and PHB (right). Log‐rank test *p*‐value measuring the global difference between survival curves and number of samples at risk in each group across time are reported. (B) Cox regression univariate analysis of OS (left) or PFS (right) data on MIF and PHB expression groups, age equal to or greater than 65 years, ISS subgroups and main molecular alterations in 489 BM MM cases for which all data were available. Number (*N*) of positive cases is indicated for each variable. Hazard Ratio, 95% confidence interval and Log‐rank *p*‐value are reported for each variable. All significant variables after BH correction are depicted in red bold.

### 3.4 Plasma PHB and MIF Levels in MM Patients

Next, we explored the potential of PHB and MIF as circulating protein biomarkers, based on the premise that tumor cells might release these proteins into circulation also within EV cargo, enabling their detection.

First, western blot analysis across five HMCL confirmed that both MIF and PHB are present in cells and matched EV, consistent with the proteomics data (Figure 6A). We next evaluated circulating levels in plasma from HD (*N* = 8/10) and newly diagnosed patients with MGUS (*N* = 10/12), SMM (*N* = 16/19) or MM (*N* = 17/19) by ELISA for MIF and PHB, respectively (Figure 6B,C). MIF concentrations were significantly higher in SMM and MM patients in comparison to HD, while MGUS did not differ significantly from controls (Figure 6B). By contrast, PHB levels did not show significant groupwise differences (Figure 6C).

**FIGURE 6:**
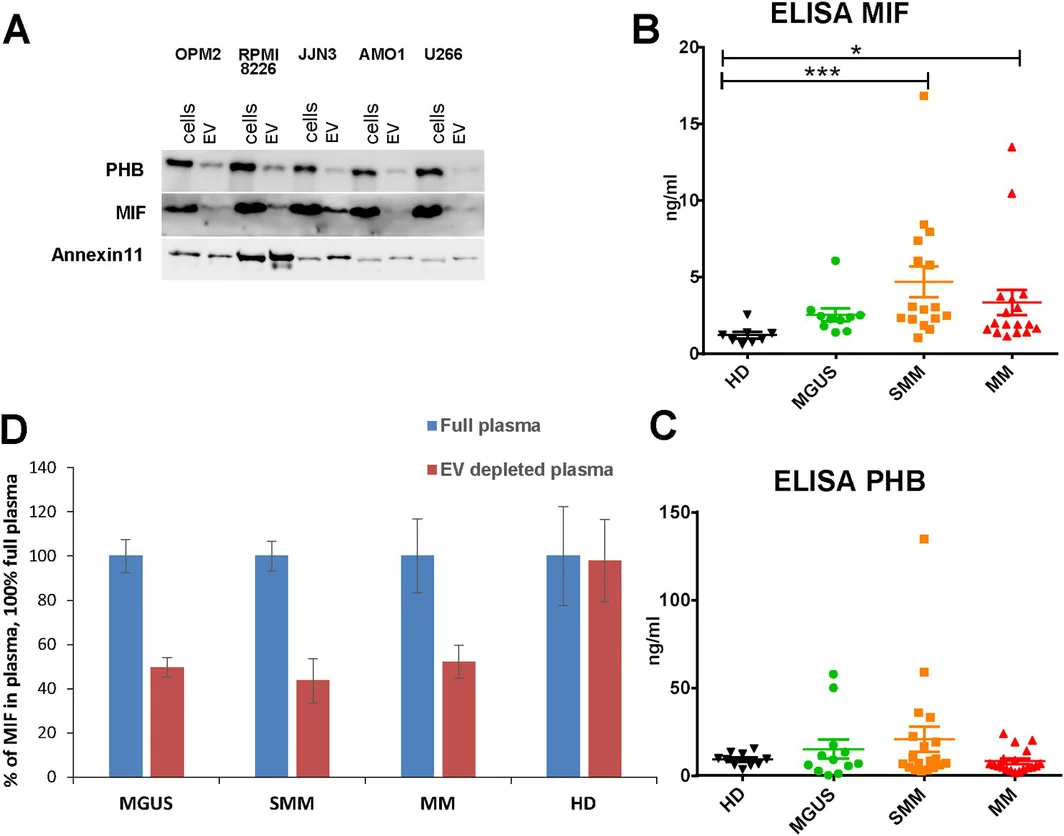
Validation of candidate EV‐related biomarkers. (A) Western blot analysis for MIF and PHB on cell and vesicular lysates from a panel of 5 MM cell lines. The level of Annexin11 was used for normalization. Twenty μg of protein lysates were loaded per lane. (B, C) ELISA for MIF and PHB on plasma derived from HD (8/10), MGUS (*N* = 10/12), SMM (*N* = 16/19), and MM (*N* = 17/19) newly diagnosed patients. Statistical analysis was performed using the Kruskal–Wallis test followed by Dunn's multiple comparisons test. *p < 0.05, ***p < 0.001. (D) ELISA of plasma depleted EV by ultracentrifugation for MIF on three independent plasma samples from patients with MGUS, SMM, MM, and HD. The results represent the percentage of MIF present in full plasma and after EV depletion by ultracentrifugation ± SEM. MIF in full plasma is settled as 100%.

Finally, to assess whether circulating MIF is associated with EV, we compared whole plasma with plasma depleted of EV by ultracentrifugation in MM patients and HD. In MGUS, MM and SMM patients, MIF plasma levels decreased by ~50% upon EV depletion, indicating that approximately half of total circulating MIF is EV associated. Notably, this reduction was not observed in healthy donors, suggesting that the EV‐bound fraction predominantly reflects tumor‐derived contributions in disease (Figure 6D). Collectively, these findings support EV‐associated MIF as a clinically noninvasive relevant biomarker for early detection and monitoring of MM and merit further prospective validation.

## 4 Discussion

EV are increasingly recognized as key players in cell‐to‐cell communication, carrying proteins, lipids, and nucleic acids that reflect the physiological and pathological state of their cells of origin. Our recent findings demonstrated that EV shed by MM cells contribute to disease progression by modulating the BM niche, promoting angiogenesis, and driving bone disease [7].

This study builds on the emerging role of EV as an important source of biomarkers and introduces a fresh approach to the identification of EV‐related biomarkers with great specificity. Given the heterogeneity of circulating EV in plasma and the fact that only a small fraction originates from tumor cells, this study adopted a strategic approach to biomarker discovery by initially focusing on EV released by MM cell lines. This allowed the identification of highly controlled and tumor‐specific vesicular proteins while minimizing contamination from non‐tumor sources such as stromal or immune cell–derived vesicles, which represent a major confounding factor when analyzing circulating EVs. However, we acknowledge that cell line–derived EVs cannot fully capture the inter‐patient and microenvironment‐driven heterogeneity of MM. This limitation is partially mitigated by the integration with large clinical datasets (MMRF CoMMpass and independent cohorts) and by the validation in patient plasma samples, which strengthen the translational relevance of our findings. Additionally, future studies should therefore extend this approach to primary MM cells and co‐culture systems that better recapitulate tumor–microenvironment interactions.

Proteomic analysis of MM‐EV released by OPM2 cell line revealed that 14% of total identified EV proteins were detectable in EV but not in the parental cells. This finding demonstrates that EV cargo does not simply mirror a proportional distribution from the cell but rather reflects a selective enrichment process. Enrichment analyses highlighted a clear distinct functional profile between EV‐associated and cell molecules. Gene Ontology analyses confirmed a functional segregation between EV‐associated set and cellular set. EV molecules are involved in extracellular communication, vesicle trafficking, and adhesion. Their localization to focal adhesions and cell–substrate junctions highlight MM‐EV as specialized mediators in intercellular signaling and with extracellular matrix interactions. In contrast, molecules enriched in parental cells were primarily linked to intracellular regulatory processes, including RNA metabolism, transcriptional regulation, and mitochondrial function, reflecting their role in maintaining cellular homeostasis. Subcellular localization analysis on the Jensen library further confirmed that EV molecules were predominantly enriched in exosome‐ and EV‐related compartments, whereas cell set was mainly associated with intracellular structures.

Importantly, through systems‐level bioinformatic analyses using the DisGeNET and TRRUST databases we show that in comparison to the cellular protein compartment, MM EV are more enriched in oncogenic pathways, transcriptional networks and disease associated proteins, positioning them as a powerful and underexploited source of tumor‐specific biomarkers. The most notable enriched transcriptional network present among cell set and further enriched in EV was that of MYC. MYC is the most commonly mutated gene in MM [36] with a crucial role in MM onset and progression [37] and an inverse correlation of its expression with PFS and OS [38]. Other oncogenic transcription networks more represented in MM‐EV than MM cells with a recognized role in MM are TWIST1 [39], SOX6 [40], SP1 [41] and SNAI1 [42]. DisGeNET database analysis revealed that MM‐EV are specifically enriched in cancer‐related pathways, whereas the cell set of MM cells exhibits a broader representation of pathways associated with various disease types mostly unrelated with cancer.

By merging discovery driven proteomics with large scale clinical datasets, we applied our flowchart to 21 candidate biomarkers that were selected through DisGeNET database analysis (Figure 7). The choice of DisGeNET as an intermediate filtering step was motivated by the aim of prioritizing proteins with established disease associations, thereby increasing the translational relevance of the identified candidates. Importantly, this approach may also enable the identification of proteins not previously associated with multiple myeloma, thus allowing the potential discovery of novel MM‐related biomarkers. Indeed, these candidates were subsequently evaluated using MM‐specific datasets, including those curated by Neri and Gutierrez, as well as the MMRF CoMMpass database, to assess their relevance in a disease‐specific context.

**FIGURE 7:**
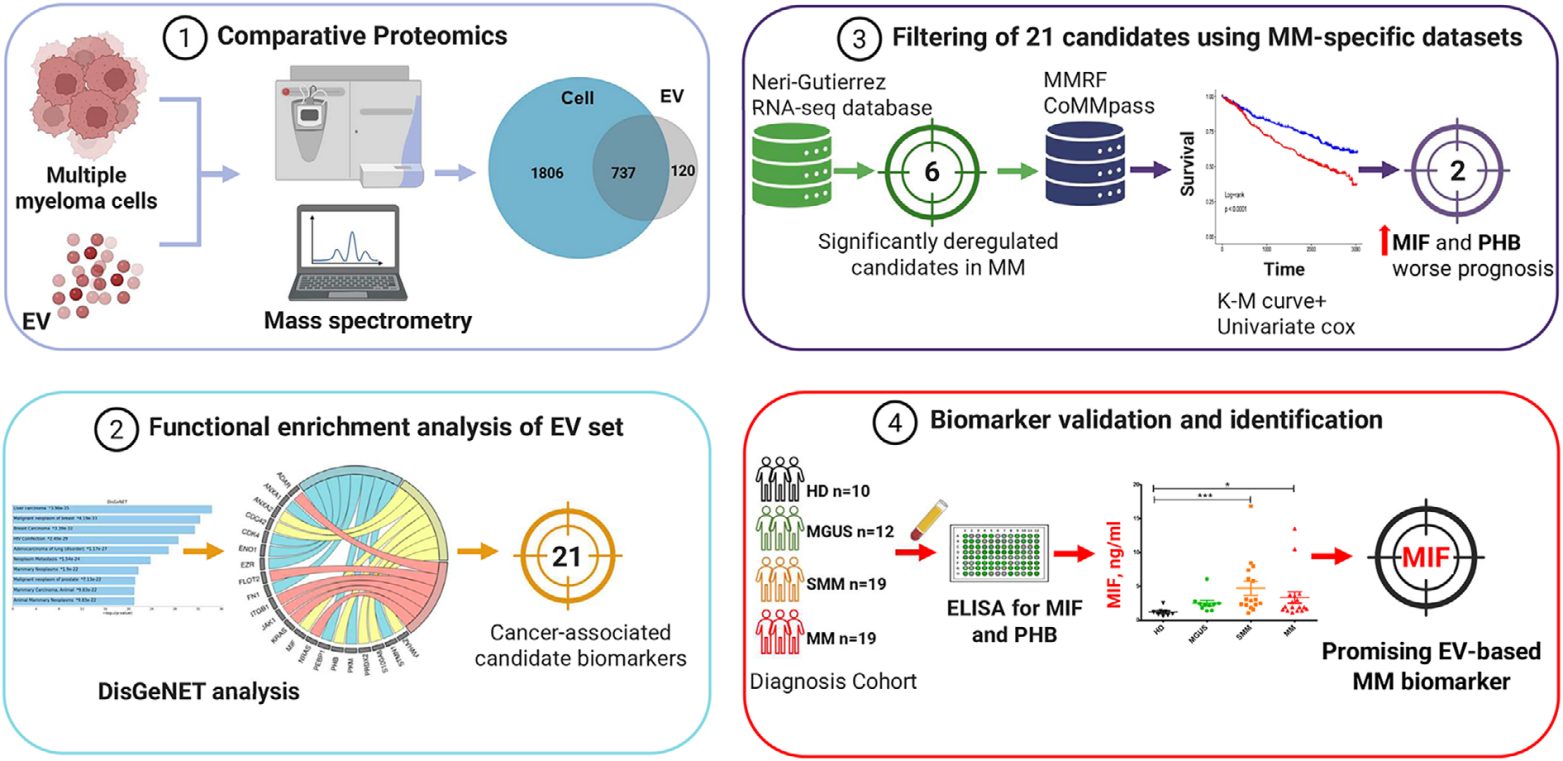
Schematic illustration of EV biomarker identification The workflow includes the following: (1) proteomic profiling of EV OPM2 versus parental cells; (2) functional enrichment and disease association analysis using DisGeNET; (3) selection of candidate proteins through MM‐specific transcriptomic datasets; and (4) biomarker validation using clinical data and patient samples identified MIF as a clinically relevant marker.

This led to the identification of MIF and PHB as candidate biomarkers that further were validated for their predictive value in plasma samples from MM patients at different disease stages. From both candidates, MIF emerged as the most clinically compelling candidate as it was significantly increased in SMM and MM patients compared to healthy donors. It suggests a potential role for MIF in disease progression and highlights its value as a biomarker for distinguishing malignant from benign/healthy conditions. Indeed, recent findings show that MIF is highly expressed in MM patients and plays a critical role in MM cell adhesion to the BM and chemotherapy response [43]. Also, Wang and colleagues indicate that MIF contributes to drug resistance by preventing drug‐induced SOD1 misfolding [44]. Moreover, in line with our results, higher MIF gene expression levels correlated with significantly shorter PFS and OS in MM patients and MIF protein expression was significantly upregulated in patients with reduced response to Bortezomib, suggesting its role as a biomarker for predicting drug response [44].

Notably, in line with our results, MIF has been reported as a predictive biomarker also in several other cancers, including prostate, ovarian and breast cancer, hepatocellular carcinoma, bladder cancer, nonmelanoma skin cancer and glioblastoma [45, 46]. Additionally, studies on glioma, pancreatic cancer and head and neck tumors have demonstrated that exosomal MIF promotes tumor growth, metastasis and drug resistance, further establishing its relevance as both a prognostic biomarker and a therapeutic target [47, 48, 49, 50].

Our preliminary data further indicate that approximately 50% of circulating MIF is associated with EV cargo, highlighting the relevance of EV‐bound MIF as a stable and specific biomarker. Interestingly, in EV‐depleted plasma from healthy donors, MIF levels were not decreased with respect to non‐depleted plasma, suggesting that only tumoral MIF is predominantly associated with EV detectable by ELISA. However, the association of MIF with EV was inferred indirectly from comparisons between whole plasma and EV‐depleted plasma rather than from direct analysis of purified EV. Further studies are therefore required to confirm its vesicular localization and to establish clinical utility in MM patient management.

In conclusion, our findings show that MM‐EV selectively package oncogenic proteins distinct from those of their parental cells, highlighting their potential role in tumor progression, intercellular communication and as a source of potential biomarkers. Our integrative approach, schematically presented in Figure 7, combines in vitro EV proteomics, multi‐omics patient cohorts and clinical sample validation providing a robust framework for EV biomarker discovery. It supports MIF as a promising tool for early detection, patient stratification and therapeutic monitoring, especially with the advancement of highly sensitive detection methods.

## Author Contributions

**Natalia Platonova:** conceptualization, investigation, data curation, writing – original draft, visualization, writing – review and editing, methodology, project administration. **Gilda Aiello:** investigation, data curation, formal analysis, writing – review and editing. **Domenica Ronchetti:** investigation, data curation, formal analysis, visualization, writing – review and editing. **Valentina Citro:** data curation, formal analysis, visualization, writing – review and editing. **Rosanna Asselta:** conceptualization, data curation, formal analysis, visualization, writing – review and editing. **Lavinia Casati:** formal analysis, writing – review and editing. **Domenica Giannandrea:** formal analysis, writing – review and editing. **Elisa Taiana:** formal analysis, writing – review and editing. **Antonino Neri:** conceptualization, writing – review and editing. **Gloria Sini:** formal analysis, resources, writing – review and editing. **Elisabetta Calzavara:** resources, writing – review and editing. **Mauro Turrini:** resources, writing – review and editing. **Alfonsina D'Amato:** conceptualization, investigation, data curation, formal analysis, writing – review and editing, methodology. **Raffaella Chiaramonte:** conceptualization, data curation, writing – original draft, visualization, writing – review and editing, funding acquisition, methodology, supervision, project administration.

## Funding

This study was supported by grants from Associazione Italiana Ricerca sul Cancro, AIRC Investigator Grant to R.C. (PI) (IG 2017 ‐ID.20614), to E.T. (PI) (MFAG 2022‐ID.27606), to D.G. (post‐doctoral fellowship type B within AIRC Investigator Grant) (20614); Italian Ministry of Research, under the complementary actions to the NRRP “D34Health Digital Driven Diagnostics, prognostics and therapeutics for sustainable heatth care” Grant (PNC0000001) to R.C. and D.G.; Università degli Studi di Milano to VC (post‐doctoral fellowship type A).

## Ethics Statement

The study was conducted according to the guidelines of the Declaration of Helsinki and approved by the Institutional Review Board of Insubria, Varese, Italy (protocol code 285/2023 and date of approval 14/06/2023).

## Consent

Informed consent was obtained from all subjects involved in the study.

## Conflicts of Interest

The authors declare no conflicts of interest.

## Supporting information


**Figure S1:** Flow‐cytometry characterization of EV markers on EVs derived from OPM2 cell line. Representative histograms show the percentage of EVs positive for CD63, CD81, and CD9, along with the MFI (Median Fluorescence Intensity) normalized to isotype controls.

**Figure S2:** Jensen compartment gene set library. It individuates associations between genes and cellular compartments for EV‐specific set (A) or for Cell specific set (B). Bars correspond to terms with significant *p*‐values (< 0.05). An asterisk (*) next to a *p*‐value denotes terms that remain significant after adjustment, with an adjusted *p*‐value < 0.05.

**Figure S3:** Cox multivariate regression analysis for MIF and PHB. Forest plot of cox regression multivariate analysis considering all features with adjusted *p*‐value < 0.05 in univariate analysis with regards to OS (A) and PFS (B) in 489 MM cases for MIF (left) and PHB (right). Hazard Ratio, 95% confidence interval and Log‐rank *p*‐value are indicated in the plot for each variable. Significant values: *p ≤ 0.05; **p ≤ 0.01; ***p ≤ 0.001.

**Table S1:** Clinical characteristics of patients at presentation.

**Table S2:** Total protein cell list.

**Table S3:** List of OPM2‐EV genes derived from DisGeNET analysis. The 21 genes selected for further bioinformatic analysis are in bold. The number of tumors in which each gene is present is indicated in brackets.

## Data Availability

The data generated during this study are included in this published article. The mass spectrometry proteomics data have been deposited to the ProteomeXchange Consortium via the PRIDE partner repository with the dataset identifier PXD063979 [21]. The analyzed MMRF CoMMpass data are available at https://research.themmrf.org/ and retrieved from the Interim Analysis 20 (MMRF_CoMMpass_IA20, accessed on 19 January 2023).
