# Supplementary material for: Protein Profile of Multiple Myeloma‐Derived Extracellular Vesicles for the Discovery of Novel Myeloma‐Related Biomarkers
Source: Cancer Sci. 2026 Jul 16;117(10):2877–90. doi: 10.1111/cas.70473 (PMC13394268; doi:10.1111/cas.70473)
Supplement: Supplementary file 1 [file CAS-117-2877-s002.docx]

Supplementary Table S3. List of OPM2-EV genes derived from DisGeNET analysis. The 21 genes selected for further bioinformatic analysis are in bold. The number of tumors in which each gene is present is indicated in brackets.

| **ANXA1 (9)** | MTAP (7) | MVP (6) | FBL (5) |
| --- | --- | --- | --- |
| **CDK4 (9)** | NCAM1 (7) | NCL (6) | FKBP4 (5) |
| **ENO1 (9)** | NME1 (7) | **NRAS (6)** | **FLOT2 (5)** |
| **EZR (9)** | NPM1 (7) | P4HB (6) | GPER1 (5) |
| **FN1 (9)** | PARP1 (7) | PA2G4 (6) | HLA-A (5) |
| **PEBP1 (9)** | PCNA (7) | PFN1 (6) | HLA-C (5) |
| **PKM (9)** | PLK1 (7) | PGK1 (6) | HNRNPA2B1 (5) |
| **STMN1 (9)** | PRKDC (7) | PPIA (6) | HSPA8 (5) |
| **ANXA2 (8)** | PTBP1 (7) | PPP2R1A (6) | IARS (5) |
| **CDC42 (8)** | RAN (7) | PRKAR1A (6) | ITGA2 (5) |
| **MIF (8)** | RELA (7) | RPS6 (6) | ITGB3 (5) |
| **PHB (8)** | RHOA (7) | RRM2 (6) | LASP1 (5) |
| **PRDX2 (8)** | RPSA (7) | S100A11 (6) | LYN (5) |
| **S100A8 (8)** | SLC2A1 (7) | SDC1 (6) | MARCKS (5) |
| **YWHAZ (8)** | TFRC (7) | SND1 (6) | MB (5) |
| ACTN4 (7) | TOP2A (7) | SRSF1 (6) | MECP2 (5) |
| AHSA1 (7) | TXNRD1 (7) | STAT1 (6) | NME2 (5) |
| AIMP2 (7) | VCP (7) | TPD52 (6) | PABPC1 (5) |
| APRT (7) | ANXA5 (6) | TPI1 (6) | PARK7 (5) |
| ARHGDIA (7) | CAPNS1 (6) | TUBA1B (6) | PPP1CA (5) |
| BSG (7) | CD151 (6) | UCHL1 (6) | PRDX6 (5) |
| CASP3 (7) | CFL1 (6) | XRCC5 (6) | PSAP (5) |
| CDH2 (7) | CKAP4 (6) | XRCC6 (6) | PTGES3 (5) |
| CIB1 (7) | DDX5 (6) | YBX1 (6) | RACGAP1 (5) |
| CLU (7) | EIF3A (6) | ACLY (5) | RAD23B (5) |
| CTAG1A (7) | EIF35 (6) | **ADAR (5)** | RAPL13 (5) |
| CTNNB1 (7) | FABP5 (6) | AIFM1 (5) | RPL19 (5) |
| EEF1A1 (7) | FLNA (6) | ANP32A (5) | RPS14 (5) |
| FASN (7) | GNAS (6) | ANXA7 (5) | RRAS2 (5) |
| FEN1 (7) | GPI (6) | ARHGEF2 (5) | RRS1 (5) |
| GAPDH (7) | H2AFX (6) | B2M (5) | SDCBP (5) |
| GNAI2 (7) | HLA-B (6) | BST2 (5) | SLC1A5 (5) |
| HNRNPA1 (7) | HMGA1 (6) | CALM2 (5) | SLC7A5 (5) |
| HSPA4 (7) | HPRT1 (6) | CALP (5) | SLC9A3R1 (5) |
| HSPB1 (7) | HSPA5 (6) | CD47 (5) | TCP1 (5) |
| INSR (7) | HSPD1 (6) | CD59 (5) | TLN1 (5) |
| **JAK1 (7)** | IGF2BP3 (6) | CLIC1 (5) | TOP1 (5) |
| KPNA2 (7) | IGF2R (6) | CLIC4 (5) | TTN (5) |
| **KRAS (7)** | **ITGB1 (6)** | DNM2 (5) | TXN (5) |
| LDHA (7) | LGALS1 (6) | EEF2 (5) | VTA1 (5) |
| LDHB (7) | LMNA (6) | EIF3E (5) | XPO1 (5) |
| MAPK1 (7) | MPO (6) | EIF4A1 (5) |  |
