# Supplementary material for: Protein Profile of Multiple Myeloma‐Derived Extracellular Vesicles for the Discovery of Novel Myeloma‐Related Biomarkers
Source: Cancer Sci. 2026 Jul 16;117(10):2877–90. doi: 10.1111/cas.70473 (PMC13394268; doi:10.1111/cas.70473)
Supplement: Supplementary file 2 [file CAS-117-2877-s005.docx]

|  | All patients | MGUS | SMM | MM |
| --- | --- | --- | --- | --- |
| Patients, no. (M/F) | 50 (25/25) | 12 (5/7) | 19 (10/9) | 19 (10/9) |
| Median age, y (IQR) | 73 (14.75) | 70.5 (18.75) | 73 (15) | 73 (10) |
| Median BM PC, % (IQR) | 25 (43) | 8.0 (0) | 22.5 (18.75) | 60.0 (21.5) |
| Median sM-protein, g/L (IQR) | 15.7 (15.3) | 11.6 (13.3) | 13.2 (7.0) | 32.0 (26.0) |
| Median uM-protein, g/24h (IQR) | 0.2 (0.60) | 0.13 (0.14) | 0.19 (0.49) | 0.60 (1.85) |
| Median sFLC, mg/L (IQR) | 189.0 (687.05) | 78.5 (226.1) | 198.75 (212.95) | 605.0 (1180.0) |
| Median B2-MG, mg/L (IQR) | 2.95 (2.0) | 2.6 (0.97) | 2.05 (1.18) | 4.03 (4.20) |
| Median calcemia, mg/dl (IQR) | 9.35 (0.70) | 9.45 (0.65) | 9.25 (0.53) | 9.55 (0.88) |
| Median WBC, x 10^9^/L (IQR) | 5.40 (2.47) | 4.75 (1.68) | 5.58 (1.90) | 5.42 (2.34) |
| Median Hb, g/L (IQR) | 130 (25.0) | 130 (9.3) | 137 (15.5) | 115.5 (46.0) |
| Median PLT, x 10^9^/L (IQR) | 230 (118) | 188 (117) | 259 (50.5) | 218 (99.5) |
| Median creatinine, (mg/dl) (IQR) | 0.8 (0.38) | 0.8 (0.25) | 0.82 (0.34) | 0.78 (0.47) |

**Supplementary Table S1. Clinical characteristics of patients at presentation**

B2-MG: B2-microglobulin; BM PC: BM plasma cells; Hb: hemoglobin; IQR: interquartile range; sM-protein: seric monoclonal protein; PLT: platelets; sFLC: involved seric free light chains; uM-protein: urinary monoclonal protein; WBC: white blood cells
