# Supplementary figures and images for: Protein Profile of Multiple Myeloma‐Derived Extracellular Vesicles for the Discovery of Novel Myeloma‐Related Biomarkers
Source: Cancer Sci. 2026 Jul 16:10.1111/cas.70473. Online ahead of print. doi: 10.1111/cas.70473 (PMC13394268; doi:10.1111/cas.70473)

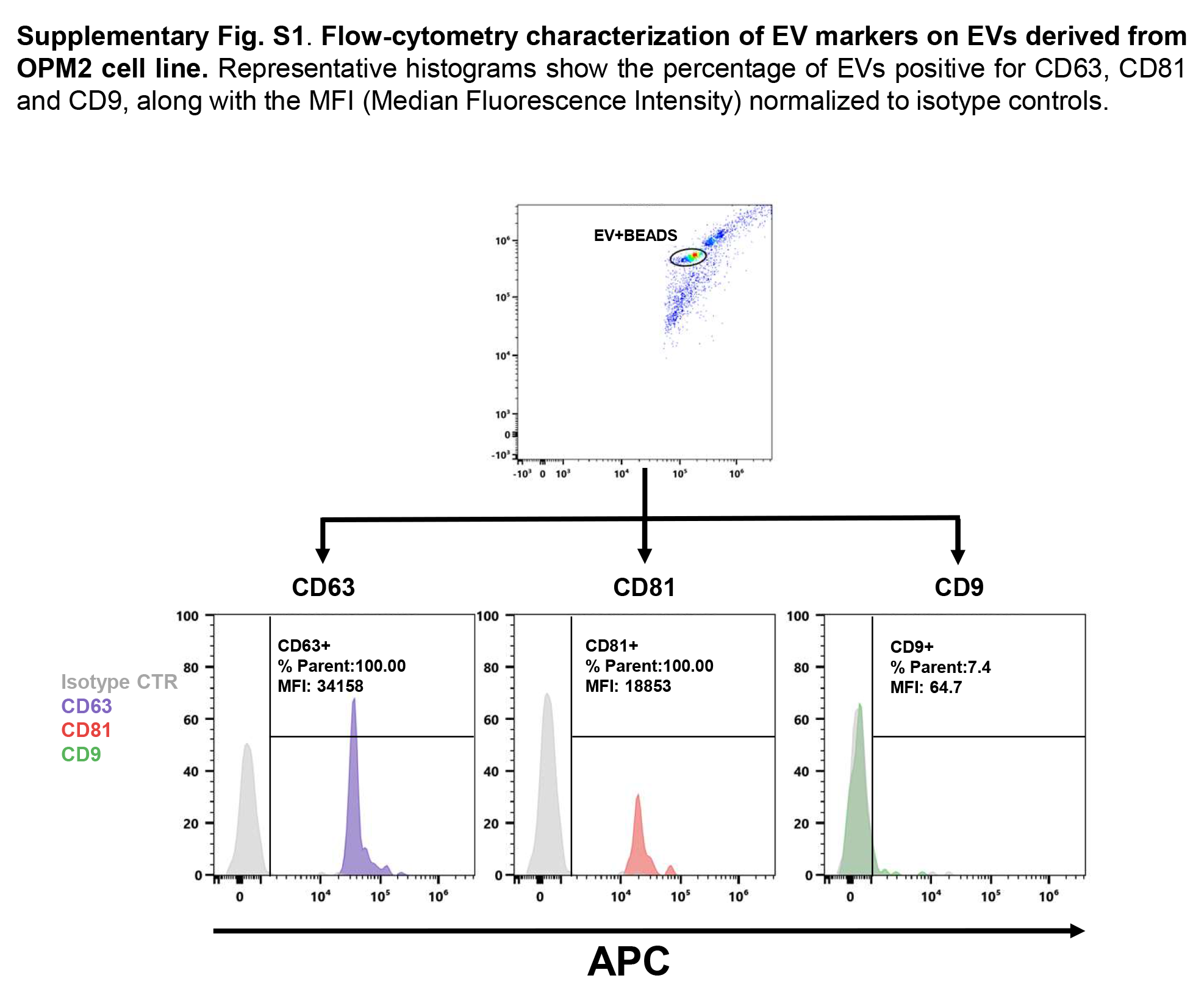

Supplement: Supplementary file 1 — Figure S1: Flow‐cytometry characterization of EV markers on EVs derived from OPM2 cell line. Representative histograms show the percentage of EVs positive for CD63, CD81, and CD9, along with the MFI (Median Fluorescence Intensity) normalized to isotype controls. [file CAS-9999-0-s003.tif]

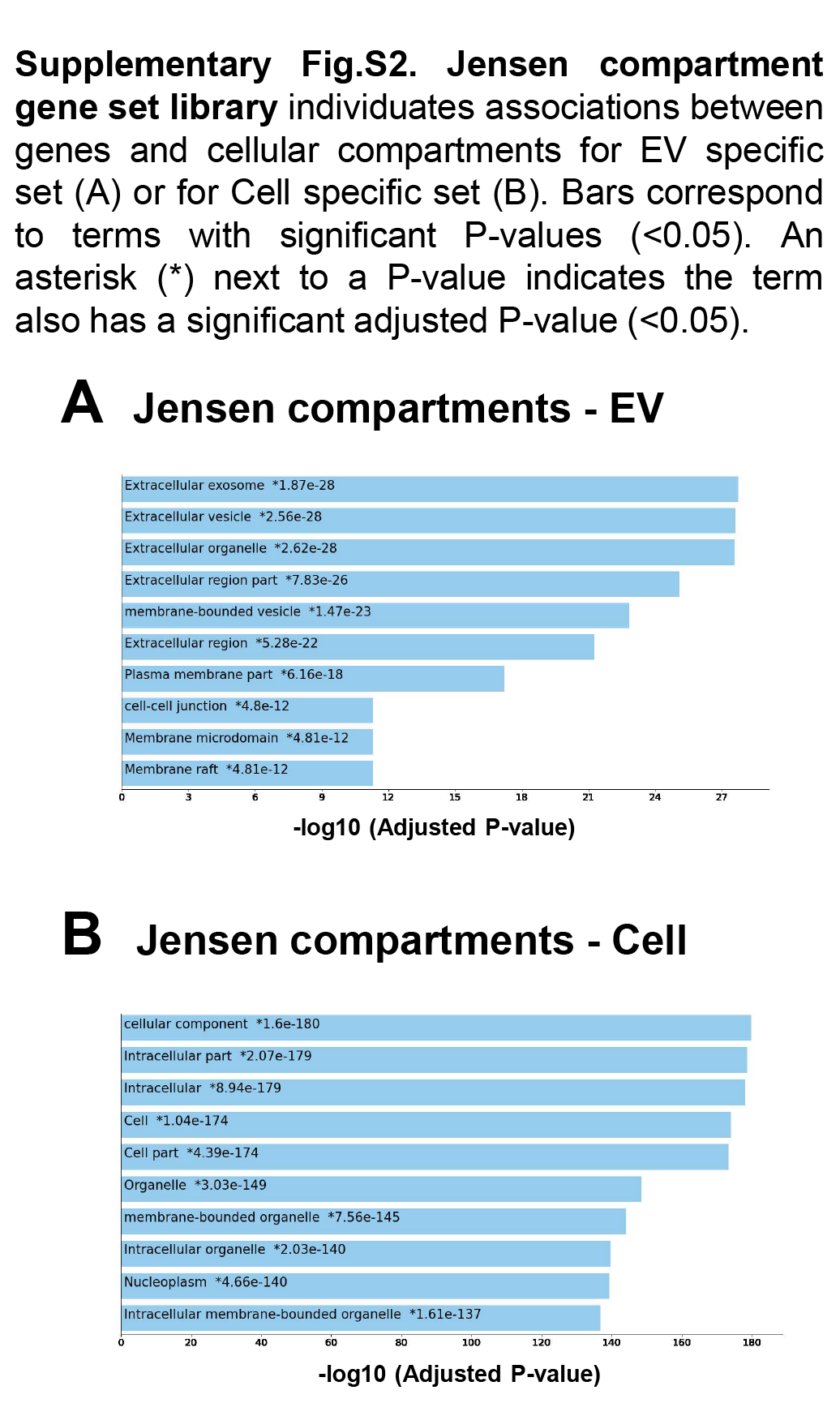

Supplement: Supplementary file 2 — Figure S2: Jensen compartment gene set library. It individuates associations between genes and cellular compartments for EV‐specific set (A) or for Cell specific set (B). Bars correspond to terms with significant p‐values (< 0.05). An asterisk (*) next to a p‐value denotes terms that remain significant after adjustment, with an adjusted p‐value < 0.05. [file CAS-9999-0-s002.tif]

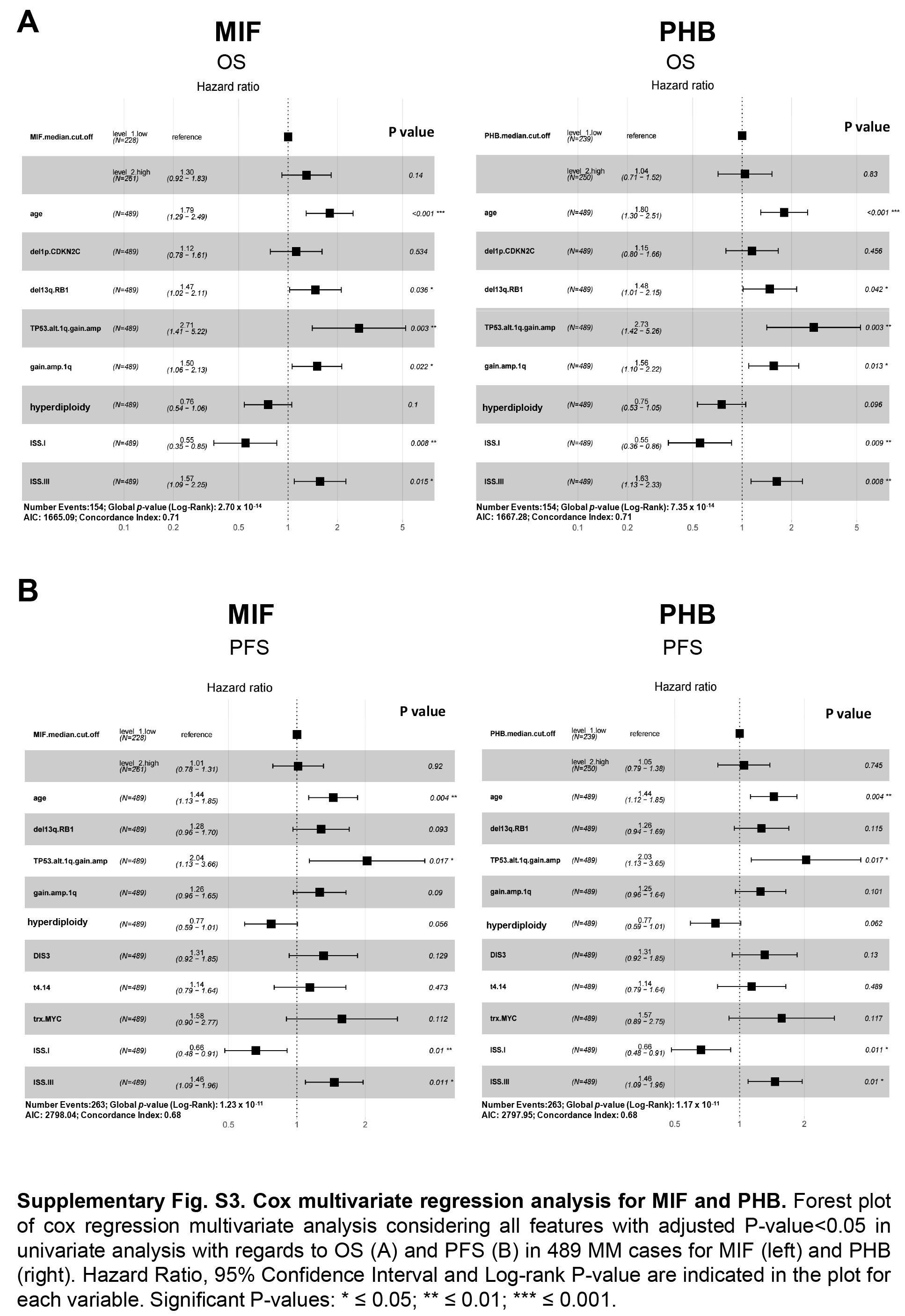

Supplement: Supplementary file 3 — Figure S3: Cox multivariate regression analysis for MIF and PHB. Forest plot of cox regression multivariate analysis considering all features with adjusted p‐value < 0.05 in univariate analysis with regards to OS (A) and PFS (B) in 489 MM cases for MIF (left) and PHB (right). Hazard Ratio, 95% confidence interval and Log‐rank p‐value are indicated in the plot for each variable. Significant values: *p ≤ 0.05; **p ≤ 0.01; ***p ≤ 0.001. [file CAS-9999-0-s005.tif]
