# Supplementary material for: Protein Profile of Multiple Myeloma‐Derived Extracellular Vesicles for the Discovery of Novel Myeloma‐Related Biomarkers
Source: Cancer Sci. 2026 Jul 16:10.1111/cas.70473. Online ahead of print. doi: 10.1111/cas.70473 (PMC13394268; doi:10.1111/cas.70473)
Supplement: Supplementary file 6 — Table S3: List of OPM2‐EV genes derived from DisGeNET analysis. The 21 genes selected for further bioinformatic analysis are in bold. The number of tumors in which each gene is present is indicated in brackets. [file CAS-9999-0-s001.docx]

Supplementary Table S3. List of OPM2-EV genes derived from DisGeNET analysis. The 21 genes selected for further bioinformatic analysis are in bold. The number of tumors in which each gene is present is indicated in brackets.

| **ANXA1 (9)** | MTAP (7) | MVP (6) | FBL (5) |
| --- | --- | --- | --- |
| **CDK4 (9)** | NCAM1 (7) | NCL (6) | FKBP4 (5) |
| **ENO1 (9)** | NME1 (7) | **NRAS (6)** | **FLOT2 (5)** |
| **EZR (9)** | NPM1 (7) | P4HB (6) | GPER1 (5) |
| **FN1 (9)** | PARP1 (7) | PA2G4 (6) | HLA-A (5) |
| **PEBP1 (9)** | PCNA (7) | PFN1 (6) | HLA-C (5) |
| **PKM (9)** | PLK1 (7) | PGK1 (6) | HNRNPA2B1 (5) |
| **STMN1 (9)** | PRKDC (7) | PPIA (6) | HSPA8 (5) |
| **ANXA2 (8)** | PTBP1 (7) | PPP2R1A (6) | IARS (5) |
| **CDC42 (8)** | RAN (7) | PRKAR1A (6) | ITGA2 (5) |
| **MIF (8)** | RELA (7) | RPS6 (6) | ITGB3 (5) |
| **PHB (8)** | RHOA (7) | RRM2 (6) | LASP1 (5) |
| **PRDX2 (8)** | RPSA (7) | S100A11 (6) | LYN (5) |
| **S100A8 (8)** | SLC2A1 (7) | SDC1 (6) | MARCKS (5) |
| **YWHAZ (8)** | TFRC (7) | SND1 (6) | MB (5) |
| ACTN4 (7) | TOP2A (7) | SRSF1 (6) | MECP2 (5) |
| AHSA1 (7) | TXNRD1 (7) | STAT1 (6) | NME2 (5) |
| AIMP2 (7) | VCP (7) | TPD52 (6) | PABPC1 (5) |
| APRT (7) | ANXA5 (6) | TPI1 (6) | PARK7 (5) |
| ARHGDIA (7) | CAPNS1 (6) | TUBA1B (6) | PPP1CA (5) |
| BSG (7) | CD151 (6) | UCHL1 (6) | PRDX6 (5) |
| CASP3 (7) | CFL1 (6) | XRCC5 (6) | PSAP (5) |
| CDH2 (7) | CKAP4 (6) | XRCC6 (6) | PTGES3 (5) |
| CIB1 (7) | DDX5 (6) | YBX1 (6) | RACGAP1 (5) |
| CLU (7) | EIF3A (6) | ACLY (5) | RAD23B (5) |
| CTAG1A (7) | EIF35 (6) | **ADAR (5)** | RAPL13 (5) |
| CTNNB1 (7) | FABP5 (6) | AIFM1 (5) | RPL19 (5) |
| EEF1A1 (7) | FLNA (6) | ANP32A (5) | RPS14 (5) |
| FASN (7) | GNAS (6) | ANXA7 (5) | RRAS2 (5) |
| FEN1 (7) | GPI (6) | ARHGEF2 (5) | RRS1 (5) |
| GAPDH (7) | H2AFX (6) | B2M (5) | SDCBP (5) |
| GNAI2 (7) | HLA-B (6) | BST2 (5) | SLC1A5 (5) |
| HNRNPA1 (7) | HMGA1 (6) | CALM2 (5) | SLC7A5 (5) |
| HSPA4 (7) | HPRT1 (6) | CALP (5) | SLC9A3R1 (5) |
| HSPB1 (7) | HSPA5 (6) | CD47 (5) | TCP1 (5) |
| INSR (7) | HSPD1 (6) | CD59 (5) | TLN1 (5) |
| **JAK1 (7)** | IGF2BP3 (6) | CLIC1 (5) | TOP1 (5) |
| KPNA2 (7) | IGF2R (6) | CLIC4 (5) | TTN (5) |
| **KRAS (7)** | **ITGB1 (6)** | DNM2 (5) | TXN (5) |
| LDHA (7) | LGALS1 (6) | EEF2 (5) | VTA1 (5) |
| LDHB (7) | LMNA (6) | EIF3E (5) | XPO1 (5) |
| MAPK1 (7) | MPO (6) | EIF4A1 (5) |  |
